# Storage of the vital metal tungsten in a dominant SCFA-producing human gut microbe *Eubacterium limosum* and implications for other gut microbes

**DOI:** 10.1128/mbio.02605-24

**Published:** 2025-03-24

**Authors:** Nana Shao, Dayong Zhou, Gerrit J. Schut, Farris L. Poole, Sydney B. Coffey, Aaron P. Donaghy, Saisuki Putumbaka, Michael P. Thorgersen, Lirong Chen, John Rose, Bi-Cheng Wang, Michael W. W. Adams

**Affiliations:** 1 Department of Biochemistry and Molecular Biology, University of Georgia 174518 , Athens, Georgia, USA; Freie Universitat Berlin, Berlin, Germany

**Keywords:** tungsten storage, human gut microbiome, Tub, TOBE, *Eubacterium limosum*, X-ray crystallography

## Abstract

Enzymes containing tungsten rather than the ubiquitous and analogous element molybdenum are prevalent in the human gut microbiome, especifically in microbes that contribute to overall gut health. *Eubacterium limosum* is a dominant human gut organism whose production of beneficial short-chain fatty acids (SCFAs) from lactate involves tungstoenzymes. Here, we characterized *E. limosum* Tub, a tungsten storage protein. Tub has a sub-nanomolar affinity for tungstate and contains a single TOBE domain first characterized in a molybdate storage protein. Crystal structures revealed Tub assembles as a hexamer composed of a trimer of dimers, capable of binding eight tungstate oxyanions at two distinct binding sites located at inter-subunit interfaces. Tungstate-saturated Tub exhibited unusually high thermal and chemical stability. Glucose-grown *E. limosum* accumulates tungsten in Tub and has low levels of two tungstoenzymes, termed WOR1 and FDH, which oxidize aldehydes and formate, respectively. Lactate-grown cells contain high concentrations of these two tungstoenzymes where WOR1 and FDH are involved in converting lactate to SCFAs. Glucose-grown cells appear to accumulate tungstate in Tub in preparation for lactate availability in the human gut. Tub and other TOBE-containing proteins are widespread in the human gut microbiome, and gene co-occurrence analysis predicts that there are comparable numbers of TOBE-containing proteins involved in the storage of tungstate as there are that bind molybdate. The results with *E. limosum* represent an important step for understanding tungsten storage mechanisms for tungstoenzymes within human gut microbes in general.

**IMPORTANCE:**

Tungsten metabolism was found to be prevalent in the human gut microbiome, which is involved in the detoxification of food and antimicrobial aldehydes, as well as in the production of beneficial SCFAs. In this study, we characterized a protein in the human gut microbe, *Eubacterium limosum*, that stores tungstate in preparation for its use in enzymes involved in SCFA generation. This revealed several families of tungstate binding proteins that are also involved in tungstate transport and tungstate-dependent regulation and are widely distributed in the human gut microbiome. Elucidating how tungsten is stored and transported in the human gut microbes contributes to our understanding of the human gut microbiome and its impact on human health.

## INTRODUCTION

The human gut microbiome has a complex taxonomic and functional composition and has been implicated in impacting a wide range of human conditions beyond digestion, such as Alzheimer’s, obesity, diabetes, and other complex diseases (1–3). The metal molybdenum (Mo) is widely used in biology, but surprisingly, the analogous group 6 element tungsten (W) has garnered increasing interest recently due to its proposed widespread utilization in the human gut microbiome (4, 5). Tungsten-containing enzymes are thought to be involved in oxidizing toxic aldehydes present in cooked foods and are generated as antimicrobials by gut microbial metabolism (4). Understanding tungsten metabolism by gut microbes could provide novel insights into human health.

All domains of life utilize molybdoenzymes (6). For example, four different types are found in human cells belonging to two of the three known molybdoenzyme families (termed SO, XO, and DMSOR [7]). Although W is best known as a Mo-antagonist, it is now becoming clear that it is more utilized in the microbial world than previously thought (8, 9). Three different ATP-dependent ABC-type transporters enable microbes to take up tungstate (WO_4_^2−^) or molybdate (MoO_4_^2−^) oxyanions. TupABC is highly specific for WO_4_^2−^ (10, 11), WtpABC transports both molybdate and tungstate (8), while ModABC is primarily known for transporting MoO_4_^2−^; it is also capable of transporting WO_4_^2−^ (12–14). Both W and Mo are incorporated into the active sites of enzymes coordinated to an organic pyranopterin cofactor. While over 50 different types of molybdoenzyme have been characterized, the only ones known to be also active with W are some examples of the DMSOR family enzymes formyl tetrahydrofuran dehydrogenase and formate dehydrogenase (FDH) (15). A fourth phylogenetically distinct family of pyranopterin-containing enzymes is known as the W-containing oxidoreductase (WOR) (4). Virtually all of them appear to use only W, as so far, there is one example where Mo might be utilized instead (16). Recent studies have revealed an abundance of genes encoding WOR enzymes and high-affinity tungstate-specific TupABC transporters within members of the human gut microbiome, suggesting the potential role of tungsten in gut microbial physiology (4, 5). Tungstate concentrations in the human gut are typically well below 100 nM (17, 18), so we were curious if a tungstate storage system, analogous to the ferritin system found for iron (19), was present in human gut microbes. However, a tungstate-binding protein with a defined physiological function has yet to be characterized from any organism.

In contrast to the situation with W, the mechanisms of Mo storage in microbes have been well studied, as Mo is a crucial component of enzymes like nitrogenase and nitrate reductase, which are ubiquitous and play key roles in the global N cycle. While some N_2_-fixing organisms contain an ATP-dependent cage-like Mo storage protein (20), most utilize members of the molbindin family (21–23), which bind the oxyanion within the transport-associated oligonucleotide/oligosaccharide binding type 1 domain (TOBE; InterPro domain IPR005116, Pfam domain PF03459). The storage protein Mop is a highly conserved 70 amino acid protein (24, 25), with the TOBE domain covering 90% of the sequence. The TOBE domain is also present in other molybdate-binding proteins such as the repressor ModE, the molybdate homeostasis regulator ModG and some forms of ModC, the ABC membrane transporter (26–28). ModE and ModG each contain two TOBE domains. ModE regulates the expression of the molybdate transporter genes (29), while ModG contributes to the synthesis of the nitrogenase MoFe-cofactor (26, 30). In *Escherichia coli,* for example, the ModC contains a single TOBE domain and several x-ray crystal structures of it are available (26, 31, 32).

Herein, we investigated W storage in the strictly anaerobic bacterium *Eubacterium limosum*. This was isolated from human feces and is a representative of the dominant Firmicutes in the gut microbiome (33). The genome of *E. limosum* encodes the ModABC transporter and a xanthine dehydrogenase-type molybdoenzyme of unknown function, but *E. limosum* also has an active tungsten-dependent metabolism (34). Its genome also contains the TupABC tungstate transporter, two different WORs (WOR1 and WOR2) and a W-containing FDH (34) and *E. limosum* preferentially takes up W rather than Mo (4). Specifically, when grown on lactate in a medium containing equal concentrations of W and Mo (100 nM), cells take up approximately 60-fold more W (6.0 ± 0.1 µmol W/g protein) than Mo (0.1 ± 0.05 µmol Mo/g protein: [34]). Moreover, W-containing WOR1 and FDH, which oxidize aldehydes and reduce CO_2_, respectively, are involved in the conversion by *E. limosum* of lactate to the short-chain fatty acids (SCFAs) acetate and butyrate, which are essential for a healthy gut microbiome (34). In this study, we present evidence for the first physiological use of a TOBE-containing protein for W storage (Tub) in a human gut microbe. Tub enables *E. limosum* to accumulate W and then supplies W for two tungstoenzymes needed for conversion of lactate to SCFAs. Moreover, TOBE-containing proteins are widespread in the human gut microbiome. We predict that in this environment, those whose physiological role is tungstate storage are comparable in number to those that function to store molybdate.

## RESULTS

### Tub stores tungstate in *E. limosum*

**Fig 1:**
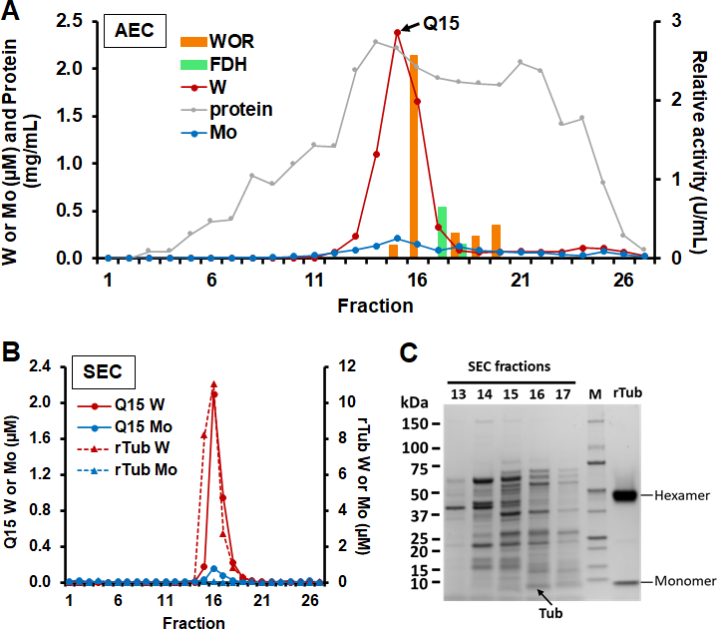
Identification of Tub within a W peak after chromatographic fractionation of *E. limosum*. The soluble cell extracts of *E. limosum* cells grown on glucose in media supplemented with 100 nM W and 100 nM Mo were fractionated using AEC (QHP anion exchange), followed by separation of the major W peak by size-exclusion chromatography (SEC). (**A**) Elution profile from QHP column indicating furfural oxidation activity (WOR, orange bars), formate dehydrogenase (FDH, green bars), protein (gray symbols), and the W (red symbols) and Mo (blue symbols) contents of fractions. The arrow at the top indicates the fraction that was further separated by SEC. (**B**) SEC column elution profiles of the major W peak fraction compared to the purified recombinant Tub (rTub) after a 1-h incubation at 4°C with tungstate. For each fraction, the W (red circle, left vertical axis) and Mo (blue circle, left vertical axis) contents are indicated for the major QHP W peak fraction. The W (red triangle and dashed line, right vertical axis) and Mo (blue triangle and dashed line, right vertical axis) contents of rTub are also indicated. (**C**) SDS-PAGE analysis of fractions 13–17 obtained from SEC column and purified rTub with both hexamer and monomer. Fractions 13–17 were treated with trichloroacetic acid (TCA) before being loaded onto the SDS gel. Molecular weights based on standards are labeled on the left. The ~7 kDa Tub band, identified with 100% coverage by LC-MS/MS, is labeled by an arrow on the bottom. All proteins identified by LC-MS/MS in fraction 16 were described in detail in Tables S1 and S2.

*E. limosum* was grown on glucose with both tungstate (100 nM) and molybdate (100 nM) added to the medium, and a cytoplasmic extract was applied to an anion-exchange column, and the W and Mo contents of the resulting fractions were analyzed using inductively coupled plasma mass spectrometry (ICP-MS). As shown in Fig. 1A, the chromatograms showed that W and not Mo were incorporated into proteins. One major tungstoprotein was observed in the fractions, with one fraction containing 2.4 µM W, compared to 0.2 µM Mo in the fraction with the highest concentration of molybdoprotein (Fig. 1A). However, the main W-protein peak was not WOR1 or FDH, as shown by the absence of aldehyde oxidation or formate-oxidizing activities, respectively (Fig. 1A). Rather, from MS analysis, the W peak originated from a 70 amino acid protein containing one TOBE-domain covering 89% of its sequence (locus tag: B2M23_RS18545) that had high similarity to the molybdate-binding protein Mop. We therefore hypothesized that this protein was involved in storing tungstate rather than molybdate. It was further purified by size-exclusion chromatography (SEC) eluting with an apparent mass of ~43 kDa that was identified by the single W peak (Fig. 1B; Fig. S1). This yielded a single protein band of ~7 kDa after sodium dodecyl sulfate-polyacrylamide gel electrophoresis (SDS-PAGE) analysis that was confirmed by MS to be the product of gene B2M23_RS18545 (100% coverage) (Fig. 1C; Table S1). We designated this protein as Tub for “tungstate-binding protein.” It is encoded by *tub* and has a calculated MW of 7,176 Da. Strep II-tagged recombinant Tub (rTub) was expressed in *E. coli*. The purified protein was also a hexamer by SEC analysis, but it did not contain significant amounts of any metal (which could have potentially been provided by *E. coli*). After incubation (1 h, 4°C) of rTub with tungstate (4× monomer excess) and SEC purification (Fig. 1B; Fig. S1) it contained 7.8 ± 0.1 g-atoms of W per hexamer and binds the equivalent of 1.3 W/monomer. A native-gel shift assay of rTub showed that it also bound molybdate and retained its oligomeric nature (Fig. S2). We therefore conclude that Tub is the major W-containing protein in glucose-grown *E. limosum* cells and binds tungstate in its hexameric form. While it also binds molybdate *in vitro*, *E. limosum* cells preferentially take up W rather than Mo (34), and its physiological role appears to be to bind tungstate. How W is differentiated from Mo at the cellular level is not at all clear at present.

### Characterization of the tungstate-binding properties of Tub

**Fig 2:**
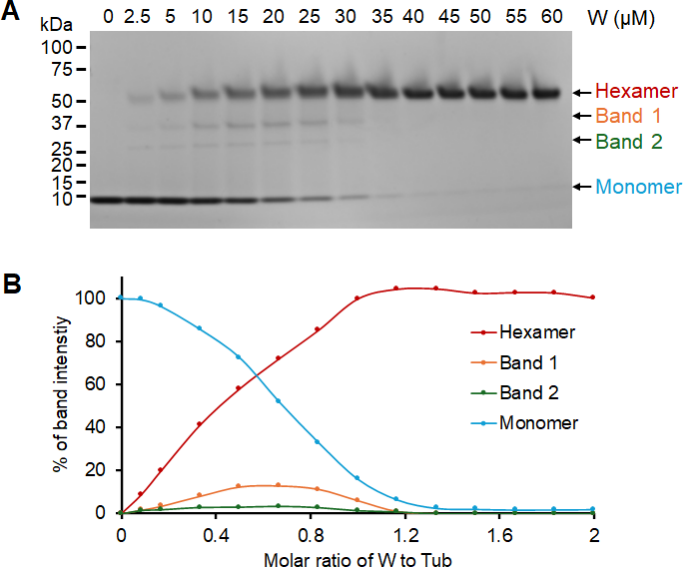
The hexameric W-saturated Tub exhibits high thermal stability. (**A**) SDS-PAGE analysis showed the hexameric form of Tub remains intact upon heating after binding to W. Numbers above the wells indicate different WO_4_^2−^ concentrations incubated with 5 µM hexameric tub. (**B**) The relative intensity of the hexamer (red), intermediate band 1 (orange), intermediate band 2 (green), and monomer (blue) in each well estimated using ImageJ (https://imagej.net/ij/).

When purified rTub (5 µM, hexamer) was incubated with tungstate, it exhibited full migration in a native mobility shift assay with ≥40 µM W, consistent with a saturating molar ratio of W to monomer of 8:1 (Fig. S3A). Surprisingly, the W-loaded protein was not denatured upon heating at 98°C for 10 min (Fig. 2A and B). Isothermal titration calorimetry (ITC) analysis revealed that Tub contains two different binding sites for tungstate (Fig. S4). Their individual binding properties could not be determined and the estimated *K*_*a*_ value was 1.00 pM with a saturating molar ratio of W to Tub of 1.44 ± 0.04:1. rTub was resistant to trypsin digestion both with and without added tungstate but was converted to the trypsin-sensitive monomeric form by TCA treatment (Fig. S5). This is consistent with the identification by MS/MS of native Tub (nTub) purified from *E. limosum* only after TCA treatment (Fig. 1C). rTub showed a similar saturation behavior with molybdate using both native mobility shift assays and ITC (Fig. S3B and S4 to S6). Similarly, Mo-loaded Tub exhibited the same thermal stability as the W-loaded form (Fig. 2; Fig. S6) and was also resistant to trypsin digestion (Fig. S5).

### The crystal structures of Tub and a proposed model for tungstate binding

**Fig 3:**
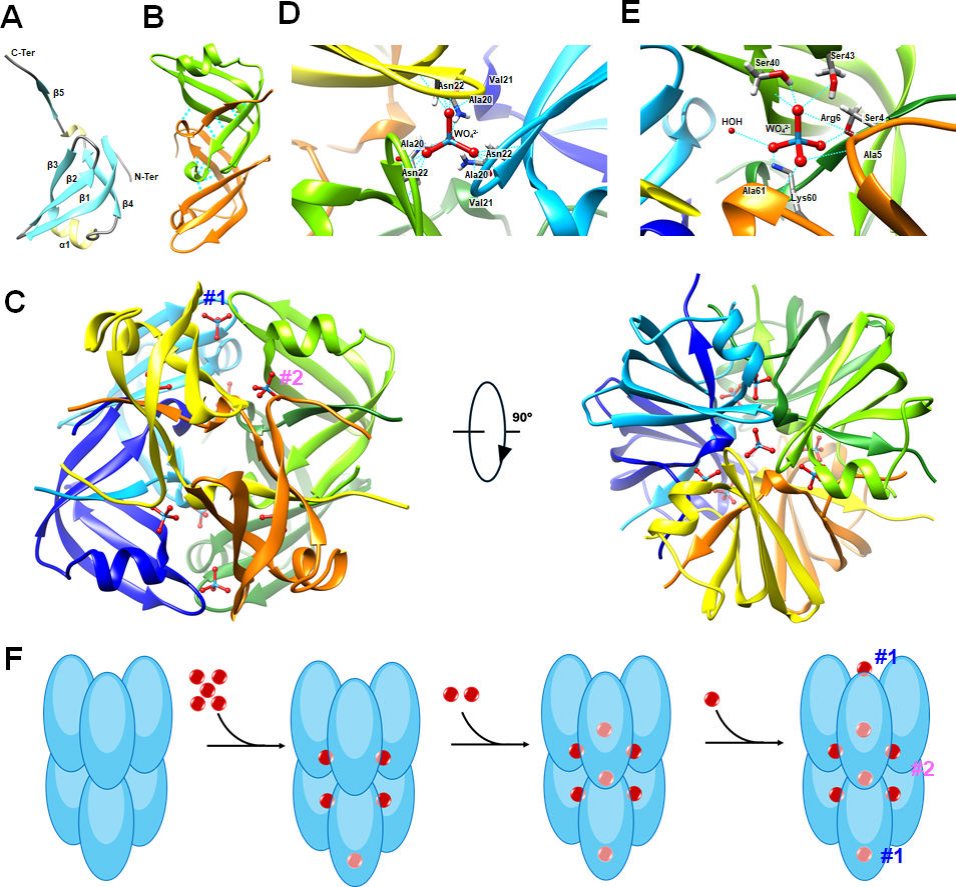
The overall structure of Tub and a proposed binding model with W. (**A**) Schematic representation of Tub monomer. The annotated secondary-structure elements are color-coded using yellow for helix, cyan for β-strand, and gray for coil. (**B**) Tub dimer is formed by a three-stranded antiparallel β-sheet, H-bonds shown as dotted blue lines. (**C**) Two orthogonal views of W-bound Tub hexamer. It comprises a trimer of dimers that binds eight WO_4_^2−^ (in ball and stick representation) at two types of binding sites: type-1 (labeled as #1 in blue) and type-2 (labeled as #2 in pink). Two type-1 sites (**D**) are positioned on a threefold NCS axis, while the remaining six type-2 sites (**E**) are found at the interfaces between two subunits. (**F**) Proposed binding model of W (red) to Tub (blue). Tub exhibits a random W-binding mechanism, where W binds to type-1 and type-2 sites simultaneously, with its final binding site located within the trimer center. Additional details are in Fig. S7.

The X-ray crystal structure of tungstate-bound rTub was determined at 1.85 Å resolution (Fig. 3; Table S3). It consists predominantly of β-strands (59.1%), where the C-terminal half of the β1, β2, and β3 form a three-stranded antiparallel β-sheet. The Tub dimer is formed by another three-stranded antiparallel β-sheet consisting of N-terminal half of β1, β4, and the β5′ from the neighboring monomer. There is also a short α-helix between β3 and β4 and a 3_10_-helical segment between β4 and β5 (Fig. 3A). In all crystal structures, three copies of the Tub dimer are arranged around a non-crystallographic threefold axis to form a spherical hexamer of 32 symmetry of 45 Å in diameter (Fig. 3B and C). Tub has eight tungstate binding sites, where two (type-1) lie at opposite poles of the hexamer with a cavity that is open to the surface (Fig. 3D). Tungstate binding involves H-bonding to the backbone of Ala20, Val21, and Asn22 from each of the three monomers along the threefold axis. The overall structure of the type-1 binding site is similar to that of the molybdate binding protein, ModG (26). The six type-2 binding sites in Tub are located at the interface of two adjacent monomers by non-crystallographic twofold symmetry producing two binding sites for every dimer (Fig. 3E). The type-2 sites are buried in the interior of the Tub hexamer with no direct connection to the solvent when tungstate is bound, which forms a complex H-bonding network involving the backbones of Arg6 and Ala61, and sidechains of Ser4 and Lys 60 from one monomer, and Ser40 backbone and sidechain from neighboring monomer. This binding site is very similar to that found in Mop and ModE (31, 32).

Additional X-ray crystal structures of rTub occupied with varying amounts of W (Fig. S7) revealed that tungstate accesses the internal type-2 binding sites through tunnels that are closed after binding. With five tungstate oxyanions bound, one tungstate binds to the type-1 site and four bind to type-2 sites. With seven tungstate bound, all six type-2 binding sites are occupied and one type-1 site. Based on the occupancy and B-factor statistics, a binding model of tungstate to Tub is proposed (Fig. 3F). Tungstate simultaneously binds to type-1 and type-2 sites indicating random binding, except for the final binding site located within the trimer center. The model is consistent with the ITC data showing that both sides have identical binding affinities (Fig. S4).

### Tub accumulates tungstate for tungstoenzymes

**Fig 4:**
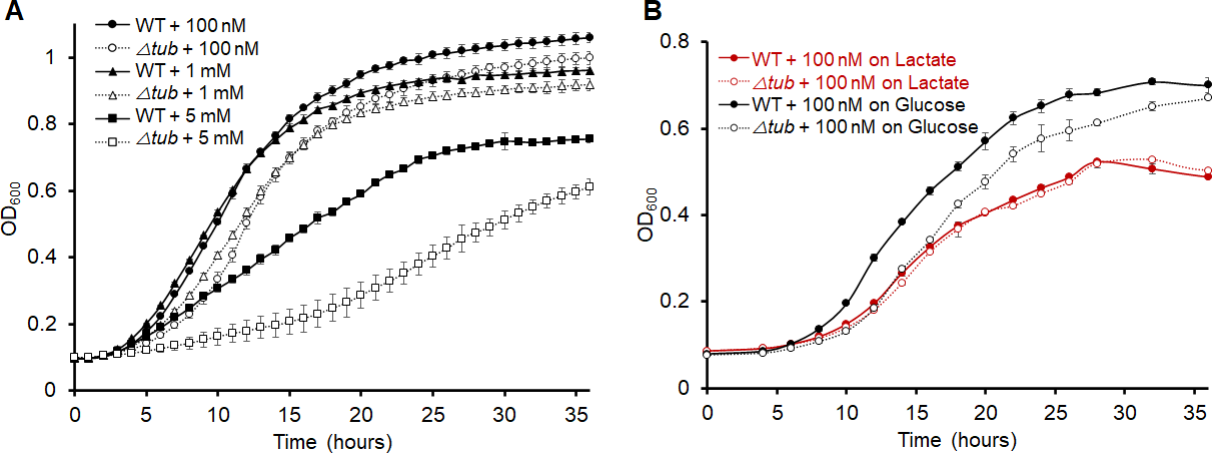
Comparison of growth between *E. limosum* wild-type and △*tub* mutant. (**A**) Growth of the wild-type and △*tub* mutant on glucose, with varying added tungstate concentrations (100 nM, 1 mM, and 5 mM), indicated by solid symbols for the wild type (circle, triangle, and square, respectively) and open symbols for the △*tub* mutant (circle, triangle, and square, respectively). (**B**) Growth curves of the wild type and △*tub* mutant on glucose (black lines) or lactate (red lines) with 100 nM W added. Averages with the standard deviations of three replicates are shown. The error bars were omitted when smaller than the data points.

We show here that Tub is the major W-containing protein in *E. limosum* when it is grown on glucose (Fig. 1A). In such cells, the tungstoenzymes WOR1 and FDH have specific activities of 1.2 and 0.3 U/mg, respectively, but these increase ~10-fold when the organism is grown on lactate (34). This increase is consistent with higher mRNA levels quantified by RT-qPCR for the genes encoding these two enzymes, which play a crucial role in the production of SCFAs from lactate by *E. limosum* (34). In contrast, the expression levels of Tub and the tungstate transporter TupABC (*tupA* was measured) do not increase significantly during growth on lactate (Fig. S8, [34]). These findings support a model in which *E. limosum* accumulates W utilizing Tub when growing on glucose, and this stored W could be used for W-dependent production of SCFAs when lactate becomes available in the human gut environment (34). To further investigate its physiological role, the *∆tub* deletion strain was constructed by established methods (35). On glucose, the mutant had a slower growth rate and grew to slightly lower densities than the parent, and this was particularly evident at non-physiological concentrations of tungstate (5 mM; Fig. 4A; Fig. S9). These results suggest that Tub may be involved in the detoxification of excess tungstate, helping maintain cellular homeostasis and supporting survival in environments with high tungstate concentrations. In contrast, during lactate growth, there was no significant difference in the growth of the two strains (Fig. 4B; Fig. S9). These data confirm the important W-accumulating role of Tub in glucose-grown cells, and in its absence, free tungstate presumably interferes with metabolism. This is not the case in lactate-grown cells where Tub does not accumulate W to the same extent due to the high expression of the two tungstoenzymes WOR1 and FDH.

When grown with either glucose or lactate as the main carbon source, *E. limosum* cells take up approximately 60-fold more W than Mo when provided at equimolar concentrations (34). Additionally, the natively purified TOBE domain-containing protein in *E. limosum* contains W rather than Mo, and thus, we can conclude that the physiological function of this protein is to store tungstate rather than molybdate, making it a Tub rather than a Mop protein. Our genomic analysis shows that *E. limosum* encodes a TupABC (for W transport) and a ModABC (for W/Mo transport), but only one TOBE domain-containing protein and no other potential W/Mo-storage proteins.

### Bioinformatic analyses of TOBE-family and tungsten-related genes in the human gut microbiome

**Fig 5:**
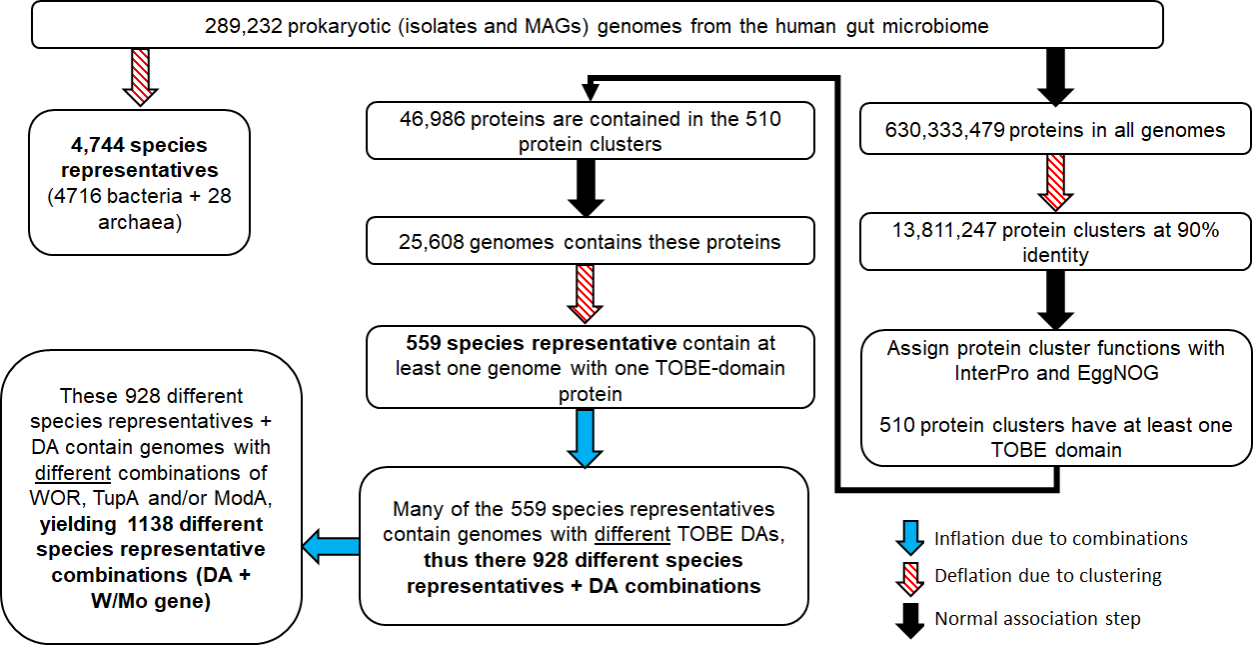
Flow chart for the analysis of TOBE domain-containing human gut species representatives using the Unified Human Gastrointestinal Genome (UHGG) data set. This flow chart illustrates the relationships between species representatives, member genomes, associated proteins, and InterPro domain architectures (DAs) in the UHGG collection.

Both Tub and Mop contain the same recognizable InterPro domain approximately 70 amino acids in length, termed the TOBE domain that binds either tungstate or molybdate depending on the protein and the physiological conditions. We investigated TOBE domain-containing proteins in the human gut microbiome databases with the goal of determining how diverse and widespread they are, and whether other TOBE domain-containing proteins, like those found in *E. limosum*, are likely to bind tungstate. As shown in Fig. 5, we mapped all occurrences of the TOBE domain within the Unified Human Gastrointestinal Genome (UHGG v2.0.2) collection that comprises 289,232 MAG and isolate nonredundant genomes (nr-genomes) and encodes 630 million proteins (36). A total of 46,986 TOBE domain-containing proteins were identified within 25,608 nr-genomes (19,673 MAGs and 5,935 isolates). We used InterPro to search for other domains contained within the TOBE domain-containing proteins, and this revealed 16 unique InterPro domain architectures (DAs). These were used to categorize the TOBE domain-containing proteins into groups likely to have physiologically related functions (Table S5) and to compare them with all TOBE domain-containing proteins found in InterPro (Table S6).

**Fig 6:**
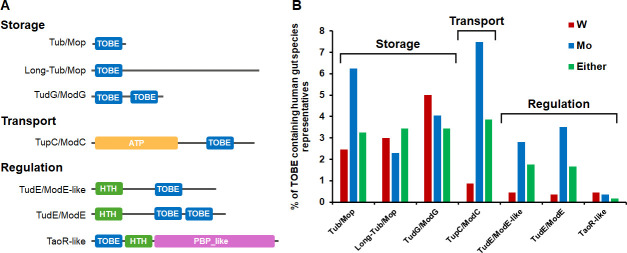
Distribution of TOBE domain-containing species representatives in human gut microbiome. (**A**) Domain architectures (DAs) of TOBE domain-containing proteins in human gut microbes highlighting different domains with distinct colors. Detailed information on representative genomes and domain InterPro entries can be found in Table S9. ATP, ABC transporter-like, ATP-binding domain; HTH in ModE/TudE or ModE/TudE-like, DNA-binding helix-turn-helix-9 domain. HTH in TaoR-like, DNA-binding helix-turn-helix-17 domain. (**B**) The percentage of species representatives with TOBE DAs classified by their predicted physiological binding of W, Mo, and either W or Mo. A W-binding (W) TOBE is predicted when TupA is present in a member genome of a species representative, Mo-binding (Mo) TOBE is predicted when only ModA is present, and W- or Mo-binding (Either) when TupA is absent but WOR is present, regardless of ModA. The percentages and groupings are calculated from the data found in Table S9.

Seven of the 16 DAs represented 94% of the TOBE domain proteins. Based on characterized members, proteins with these DAs are predicted to serve one of three primary roles related to W or Mo, either storage (28%), transport (28%), or regulation (38%) (Fig. 6A; Table S5) (37). There are three major DAs involved in W/Mo storage. First is the Tub/Mop proteins that include *E. limosum* Tub and the previously characterized Mop (32, 38, 39). In addition, there is a previously unreported longer version (>75 total amino acids) of Tub/Mop that we designate Long-Tub/Mop, also predicted to be involved in W/Mo storage (Fig. 6A). The third storage DA is TudG/ModG, where TudG is the W-binding homolog. Trimeric ModG binds eight molybdates and is involved in intracellular Mo homeostasis and nitrogenase synthesis (26, 30). Only one major DA is involved in W/Mo transport, and this encompasses ATP-dependent TupABC and ModABC where the C subunit contains the TOBE domain (Fig. 6A). There are three major DAs involved in regulation. Two are ModE homologs (TudE is predicted to use W) that contain a helix-turn-helix DNA-binding domain in addition to either one (TudE/ModE-like) or two (TudE/ModE) TOBE domains (Fig. 6A). The third regulatory DA is named TaoR-like because it contains the canonical HTH and PBP-like domains of TaoR in addition to the TOBE domain (Fig. 6A). TaoR is a characterized regulator of the tungsten-containing aldehyde oxidoreductase (40).

The UHGG collection from the gut microbiome (289,232 genomes) has a large number of highly similar MAG and isolate genomes. To remove bias and simplify analysis, these genomes were collapsed into 4,744 UHGG species representatives (where there are often hundreds or thousands of member genomes per species representative) to which the TOBE domain-containing proteins and other W/Mo related proteins (TupA, ModA, and WOR) were mapped (Fig. 5; Tables S5 and S7 to S10). About ~12% (559) of species representatives have at least one member genome with at least one TOBE domain-containing protein (Table S7). Of these species representatives, half (48%) have a member genome with at least two TOBE proteins, with some genomes contain as many as five TOBE proteins (Table S7). Similarly, TupA was found in 329 out of 4,744 species representatives, ModA in 766 out of 4,744, and WOR in 490 out of 4,744 (Table S8). To estimate how many TOBE domain-containing proteins are likely to bind W rather than Mo, the species representative genomes were split into four groups based on their content of other Mo/W related genes (ModA, TupA, and WOR) (Table S9). Specifically, a W-binding TOBE is predicted when TupA is present in a member genome of a species representative, a Mo-binding TOBE is predicted when only ModA is present, and a W- or Mo-binding is predicted when TupA is absent but WOR is present. Finally, there is a group of TOBE domain-containing species representatives where no member genome is predicted to contain TupA, ModA, or WOR—these are classified as unknowns. All seven common TOBE DAs involved in storage, transport, and regulation are predicted to have tungstate binding versions based on the presence of other tungsten-related genes in the genome. Importantly, the number of species representatives containing TOBE DAs that are predicted to use W is similar to those that are predicted to use Mo (Fig. 6B).

## DISCUSSION

In general, the TOBE-family of proteins has been characterized as being involved in intracellular molybdate storage and homeostasis. Indeed, there is only one example of a Mop protein binding tungstate, but its physiological role is unclear (41). Herein, the function of a TOBE-family protein from the human gut microorganism *E. limosum* is shown to be tungstate storage, even when the organism is grown in the presence of tungstate and molybdate (each 100 nM). Tub demonstrates comparable binding behavior for both tungstate and molybdate *in vitro*. Furthermore, Tub exhibits a much higher binding affinity (*K*_*a*_ ~1.0 pM for both type-1 and type-2 sites) for these anions than those reported for molybdate-binding TOBE family members, such as Mop, ModE, and ModG. For instance, Mop from *Haemophilus influenzae* exhibits a *K*_*a*_ of ~50 nM in type-1 site and ~11.8 nM in type-2 site (39) and MopII from *Clostridium pasteurianum* has a molybdate binding *K*_*a*_ of ~0.2 µM in type-1 site and ~4.8 µM in type-2 site (32). The ModE regulator has an affinity of ~0.8 µM (42), while ModA displays an affinity of 2–27 nM (12). We hypothesize that *E. limosum* Tub receives W directly from the tungsten transporter TupABC. The extracellular TupA of TupABC binds tungstate with an affinity of ~0.5 nM (43) and releases it *in vivo* upon interaction with TupBC in the cytoplasmic membrane using ATP hydrolysis as the energy source (43, 44). Some ModC and TupC proteins contain a single C-terminal TOBE domain, but a dimer of the TOBE domains would be required for Mo or W binding (37). This could be achieved because ModBC is a homodimer of heterodimers in the membrane as ModB_2_C_2_ (45). How Tub releases tungstate for incorporation into the pyranopterin cofactor in WOR and FDH is not clear. In molybdopyranopterin biosynthesis, ModE contains an N-terminal helix-turn-helix DNA-binding domain and two TOBE domains (Fig. 6A) and functions as a transcriptional repressor for *modABCD* and an activator of the *moaABCED* operon (46), and we propose that an analogous system is present for tungsten (Fig. 6B). In any event, we demonstrate that *E. limosum* accumulates W in Tub when grown on glucose, likely in preparation for the dramatic increase in tungstoenzyme synthesis (WOR1 and FDH) when lactate becomes available, a dynamic situation readily encountered in the gut.

Our bioinformatic analysis showed that TOBE-containing proteins that bind tungstate or molybdate are widespread and conserved in the human gut microbes, grouping into seven major TOBE-containing DAs involved in the storage, transport, and regulation of these oxyanions (Tables S9 and S10). Whether or not a TOBE protein binds molybdate or tungstate cannot be determined by sequence or structural information alone. However, as we show here, the presence of other W- or Mo-related genes present in the genome can provide insight into the identity of the physiologically relevant metal for the TOBE protein. For example, no TupABC homolog has been characterized as transporting a significant amount of molybdate (43, 44). The same cannot be said for ModABC or WtpABC, which transport both oxyanions (43, 44). A transport system specific for tungstate is probably needed for W-utilizing microbes because W is far less abundant than Mo in most environments and is required for specific enzymatic reactions with low reduction potentials. In fact, recent studies have uncovered FDH and WOR enzymes that use W in human gut microbes (34). Therefore, we suspect these gut microbes may utilize a mechanism to store precious W just like Mo is stored in N_2_-fixing microbes.

We categorized UHGG species representatives as using W, Mo, or either based on their content of TupA, WOR, and/or ModA and then mapped the TOBE domain-containing proteins along with their DAs onto these genomes (Fig. 6B; Table S9). As a whole, there are comparable numbers of W and Mo using species representatives containing storage TOBE DAs (Fig. 6B). In fact, the Long-Tup/Mop and TudG/ModG DAs are found in more species representatives predicted to use W rather than Mo. In contrast, the transport (TupC/ModC) and two regulatory (TudE/ModE-like and TudE/ModE) DAs are predicted to predominantly be located in Mo- rather than W-utilizing species representatives (Fig. 6B). In general, we conclude that TOBE domain-containing proteins are not only widespread in human gut microorganisms, but perform a diversity of functions in Mo/W storage, transport, and regulation. The main result herein identifying Tub as a tungstate rather than a molybdate storage protein in *E. limosum* likely has broader implications with diverse TOBE domain-containing proteins binding tungstate in other predominantly W-using human gut microorganisms.

## MATERIALS AND METHODS

### Growth of *E. limosum*

*E. limosum* ATCC 8486 was obtained from the American Type Culture Collection. The growth medium for *E. limosum* contained 0.5 g/L yeast extract, 1 g/L cysteine, 10 mM 3-(N-morpholino)propanesulfonic acid (MOPS), 10 mM acetate, and 1 mM KH_2_PO_4_, along with salts, a vitamin mixture and trace elements as previously described (4). The cells were cultured at 35°C on either glucose (28 mM) or lactate (70 mM) with the indicated concentration of (NH_4_)_2_MoO_4_·4H_2_O and/or Na_2_WO_4_·2H_2_O. They were grown in 160 mL serum bottles containing 50 mL base medium shaking at 40 rpm under N_2_/CO_2_ (4:1, vol/vol), in 300 µL wells in 96-well plates with shaking at 100 rpm (Vantastar, BMG lab-tech, Ortenberg, Germany) placed in an anaerobic chamber with a 95% N_2_/CO_2_ (4:1, vol/vol) and 5% H_2_ atmosphere, or in a 20 L fermenter with stirring at 150 rpm and sparging with N_2_/CO_2_ (4:1, vol/vol) at a rate of 1.5 L/min. The cells were harvested by centrifugation (10,000 × *g* in a continuous flow Sharples centrifuge) and then frozen in liquid nitrogen and stored at −80°C until use.

### Purification and identification of native Tub

All purification steps were performed anaerobically either in a Coy anaerobic chamber (95% Ar and 5% H_2_) or using sealed serum bottles with an Ar headspace. Frozen fermenter-grown *E. limosum* cells (10 g wet weight) were thawed and resuspended in 40 mL of lysis buffer containing 50 mM HEPES, pH 7.5, 5% trehalose (wt/vol), 1 mM cysteine, 0.1 mg/mL DNase I, and Protease Inhibitor Cocktail Tablets (Roche, New York, MO, USA). The cells were lysed by sonication on ice, followed by centrifugation at 35,000 × *g* for 1 h at 10°C. The soluble extract was applied to a 5 mL Q Sepharose high-performance (QHP) custom column pre-equilibrated with 25 mM HEPES, pH 7.5, 1% trehalose (wt/vol), and 1 mM cysteine, and bound proteins were eluted using a linear gradient NaCl (0–1 M) in the same buffer. Fractions containing W were concentrated using a 10 kDa cutoff filter (Millipore) and purified with a Bio-Rad Enrich SEC 650 (10 × 300) column equilibrated with 25 mM Tris-HCl, pH 7.6, 100 mM NaCl. Protein concentrations were determined with the Bradford reagent (Bio-Rad) and Mo and W were measured by ICP-MS as described previously (4). Furfural oxidation (WOR) and FDH activities were measured under anaerobic conditions at 35°C as previously described (4).

### Expression and purification of recombinant Tub

The Tub encoding gene *tub* (locus tag: B2M23_RS18545) was cloned into pET-24a(+) plasmid (Novagen) with a C-terminal Strep II tag under the control of T7 promoter. The primers are listed in Table S11. The expression vector was transformed into *E. coli* Rosetta BL21(DE3) (Novagen). The single-colony transformant was cultured in 2 L LB medium with 50 µg/mL kanamycin at 37°C with rotation at 220 rpm until reaching an *A*_600_ of 0.6. The medium was supplemented with 0.5 mM isopropyl β-d-1-thiogalactopyranoside (IPTG) to induce overnight production of rTub at 25°C with rotation at 180 rpm. The cells were harvested by centrifugation at 7,000 × *g* for 10 min at 4°C and transferred to a Coy anaerobic chamber (95% Ar and 5% H_2_). All subsequent purification steps were performed anaerobically. The cells were resuspended in 100 mM Tris-HCl, pH 7.6, 100 mM NaCl, and lysed by sonication in the presence of 0.5 mg/mL lysozyme and 0.1 mg/mL DNase I. The cell lysate was centrifuged at 35,000 × *g* for 1 h at 10°C. The supernatant was mixed with 5 mL of Strep-Tactin Superflow Plus resin (IBA Lifesciences, Göttingen, Germany) equilibrated with the same buffer, incubated at 4°C for 1 h with shaking, and applied to a 20 mL gravity flow column. Proteins were eluted with the same buffer containing 2.5  mM desthiobiotin. Fractions containing rTub were concentrated using an Amicon Ultra centrifugal filter (Millipore, 10 kDa molecular weight cutoff) in 25 mM Tris-HCl, pH 7.6, and 100 mM NaCl. rTub was quantitated at 280 nm using an estimated extinction coefficient (6,990 M^−1^ cm^−1^) based on the amino acid composition (47). For trichloroacetic acid (TCA) precipitation, 1 vol of 28% (wt/vol) TCA was added to 4 volumes of purified rTub and incubated at 4°C for 10 min. The sample was centrifuged at 12,000 × *g* for 5 min and the supernatant was removed. The protein pellet was washed two times with ice-cold acetone, followed by centrifugation at 12,000 × *g* for 5 min each time. The final pellet was dried at 95°C for 5–10 min to evaporate the acetone. For MS analysis, purified rTub was precipitated with TCA, washed two times with acetone, resuspended in loading buffer (Bio-Rad), and loaded onto precast 4–15% SDS-PAGE gels (Bio-Rad). Following staining with AcquaStain (Bulldog Bio), gel bands were digested by trypsin overnight at 37°C and analyzed using a Bruker Autoflex Matrix-Assisted Laser Desorption Ionization Time-of-Flight mass spectrometer. For native Tub, the SDS-PAGE gel band was digested by trypsin and analyzed by liquid chromatography with tandem mass spectrometry (LC-MS/MS) using a Thermo Fisher LTQ Orbitrap Elite Mass Spectrometer coupled with a Proxeon Easy NanoLC system (Waltham, MA, USA).

### Knockout strain construction

To delete *tub* (locus tag: B2M23_RS18545) from *E. limosum* (35), linear DNA fragments were assembled using NEBuilder HiFi assembly master mix. It contained *ermB* flanked by ~1,000 bp regions homologous to the upstream and downstream of the *tub* gene. The primers used are listed in Table S11. Fragments were transformed into wild-type ATCC 8486 following a modified protocol (48, 49). For transformation, 5 µL linear DNA was cleaned by the Zymo DNA, and transformants were plated on RCM plates containing 1 µg/mL clarithromycin. Colonies containing the deletion strain were confirmed by colony PCR and sequencing.

### Protein crystallization and structure determination

Crystals of Tub were grown by hanging drop vapor diffusion at 20°C using 2 µL drops containing equal volumes of the protein solution (15–25 mg/mL) and crystallization cocktail (summarized in Table S3). Crystals were harvested, cryoprotected, and then flash cooled to 100 K for data collection. Diffraction data were collected on the Rigaku XtalLab system with a microfocus X-ray generator (CuKα) and a Pilatus 200K detector at 100 K. Typically, a total of 1,440 images covering 360° were recorded for each crystal. The diffraction data sets were then indexed, integrated, and scaled using HKL-3000 (50). For tungstate-bound Tub structures, phase information was obtained using single-wavelength anomalous diffraction (SAD). Heavy atom search, W-SAD phasing, density modification, and model building were carried out using Phenix v1.12 (51). The crystal structure of apo-Tub was determined by the molecular replacement (MR) method using the tungstate-bound Tub model as the search model. The Tub models were further refined by manual examination of the electron density maps and rebuilding using Coot (52), followed by refinement. Data collection and refinement statistics are summarized in Table S4. The Tub coordinates and structure factors have been deposited in the Protein Data Bank with the PDB accession numbers 9BEB (eight tungstates bound), 9BEM (seven tungstates bound), 9BEL (five tungstates bound), and 9BJF for apo-Tub.

### Bioinformatics analysis

The analysis of all known TOBE domain-containing proteins and architectures was performed using the InterPro DA web tool (InterPro, https://www.ebi.ac.uk/interpro/search/ida/) in May 2024. In the human gut, the UHGG data set version 2.0.2 identified 289,232 prokaryotic genomes (isolates and MAGs) from the human gut microbiome and clustered them into 4,744 species representatives (4,716 bacteria + 28 archaea) (53). The approximately 630 million protein sequences from the 289,232 genomes were clustered at 90% amino acid identity (UHGP-90) into 13,811,247 protein clusters. These protein clusters were then searched using InterProScan (v5.39-77.0) and EggNOG mapper (emapper-2.1.3 [53]). The UHGP-90 InterPro and EggNOG data sets were searched for the following IPR numbers and COG numbers based on their annotations and ability to accurately classify proteins with a characterized function. Any UHGP-90 proteins that matched any of these ID numbers with *e*-values ≤1E−10 were selected, along with all their other InterPro and EggNOG matches, and then the protein was mapped back to one of the 4,744 species clusters. These ID number are as follows: TOBE domain (IPR005116), DNA-binding helix-turn-helix #17 (IPR041657), DNA-binding helix-turn-helix #1 (IPR000847), DNA-binding helix-turn-helix #3 (IPR001387), periplasmic binding domain (IPR024370), TupA-like (IPR024370) and ModA (COG0725), TupA (COG2998), and WOR (IPR013983 and IPR001203). The InterPro periplasmic binding domain (IPR024370) matches TupA but is not specific to TupA; however, the EggNOG COG2998 is very specific for characterized TupA proteins, and thus, it was used to classify TupA proteins instead of IPR024370. The data were parsed and analyzed using custom Perl scripts and a relational database.

## Data Availability

All data generated or analyzed during this study are included within the paper and supporting information. The atomic coordinates have been deposited in the Protein Data Bank (www.pdb.org) under the PDB ID codes 9BEB, 9BEM, 9BEL, and 9BJF.

## SUPPLEMENTAL MATERIAL

Supplemental material mbio.02605-24-s0001.pdf Supplemental figures and tables.

Table S1 mbio.02605-24-s0002.xlsx Proteins identified by LC-MS/MS in the ~7-kDa SDS gel band of fraction 16 from SEC column.

Table S2 mbio.02605-24-s0003.xlsx Proteins identified by LC-MS/MS in fraction 16 from SEC column.

Table S10 mbio.02605-24-s0004.xlsx UHGG genes, genomes, and species representative that encode a TOBE domain protein.

ASM does not own the copyrights to Supplemental Material that may be linked to, or accessed through, an article. The authors have granted ASM a non-exclusive, world-wide license to publish the Supplemental Material files. Please contact the corresponding author directly for reuse.
