# Supplementary material for: Storage of the vital metal tungsten in a dominant SCFA-producing human gut microbe Eubacterium limosum and implications for other gut microbes
Source: mBio. 2025 Mar 24;16(4):e02605-24. doi: 10.1128/mbio.02605-24 (PMC11980592; doi:10.1128/mbio.02605-24)
Supplement: Supplemental material — Supplemental figures and tables. [file mbio.02605-24-s0001.pdf]

**Supporting Information**

# Storage of the Vital Metal Tungsten in a Dominant SCFA-Producing Human Gut Microbe *Eubacterium limosum* and Implications for Other Gut Microbes

Nana Shao, Dayong Zhou, Gerrit J. Schut, Farris L. Poole, Sydney B. Coffey, Aaron P. Donaghy, Saisuki Putumbaka, Michael P. Thorgersen, Lirong Chen, John Rose, Bi-Cheng Wang and Michael W. W. Adams*

Department of Biochemistry & Molecular Biology, University of Georgia,

Athens, GA 30602, USA

**Supplementary Figures S1-S9**

**Supplementary Table S1-S11**

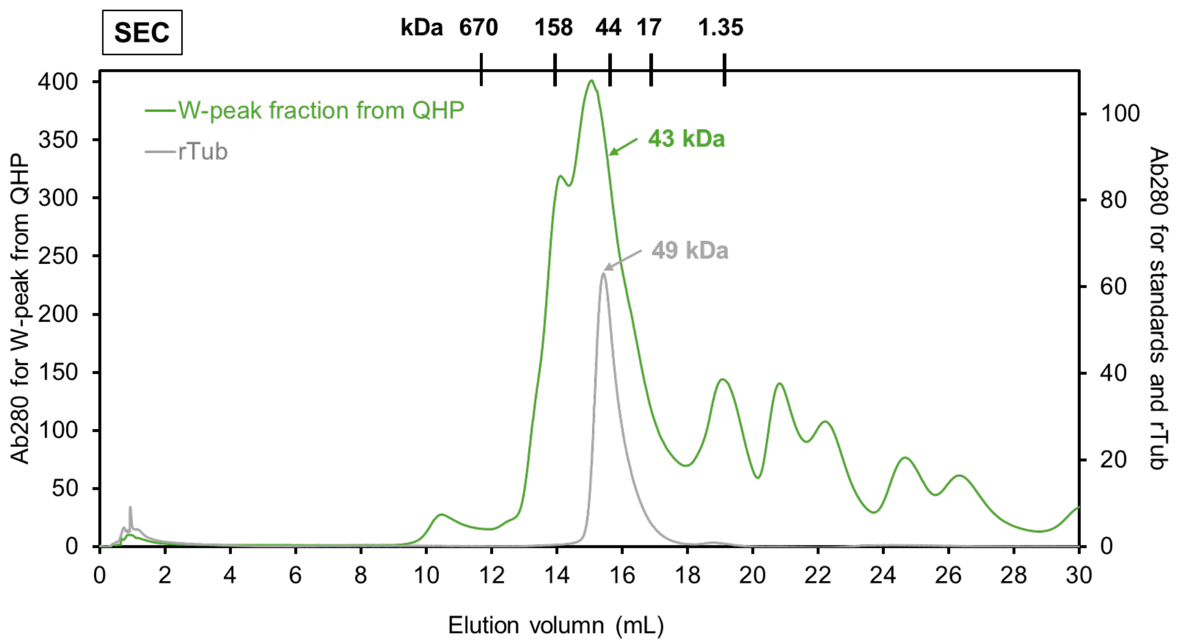


**Fig. S1. Fractionation of the peak W-containing fraction from the QHP column by size-exclusion chromatography reveals Tub is a hexamer.** The major W peak fraction from QHP column (see Figure 1A) was fractionated by SEC (green chromatogram). Purified recombinant Tub (rTub, in gray) was fractionated on the same SEC column as a control. The retention volumes of the molecular markers are shown. Native Tub has a MW of 43 kDa but rTub is larger (49 kDa) due to its affinity tag.

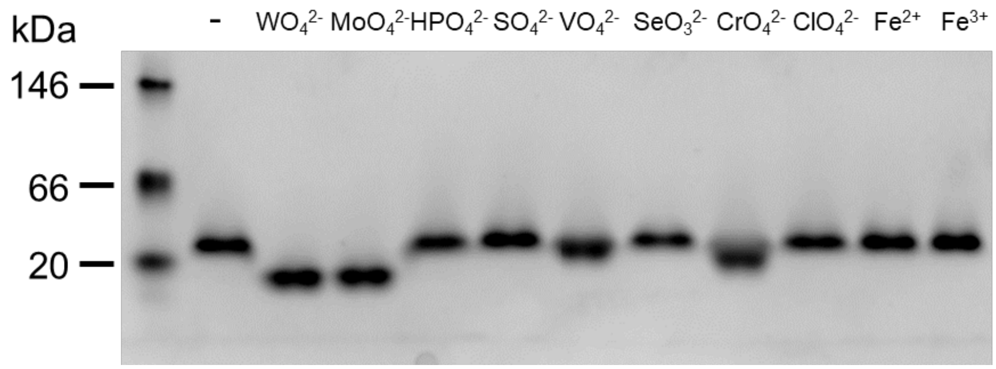


**Fig. S2. Metal oxyanions and cations binding to Tub as shown by native mobility shift assay.** The native gel-shift assays were performed following the methods developed by Rech *et al*. (1996) (1). Purified Tub aliquots (20 μM) were incubated on ice for 1 h with 400 μM of the indicated metal. The incubated samples were mixed with the native loading buffer (Bio-Rad) and loaded onto a 7.5% native polyacrylamide gel (Bio-Rad). The gel was run in a buffer containing 25 mM Tris and 192 mM glycine at 4 °C for 75 minutes at 100 V, and then stained with AcquaStain (Bulldog Bio).

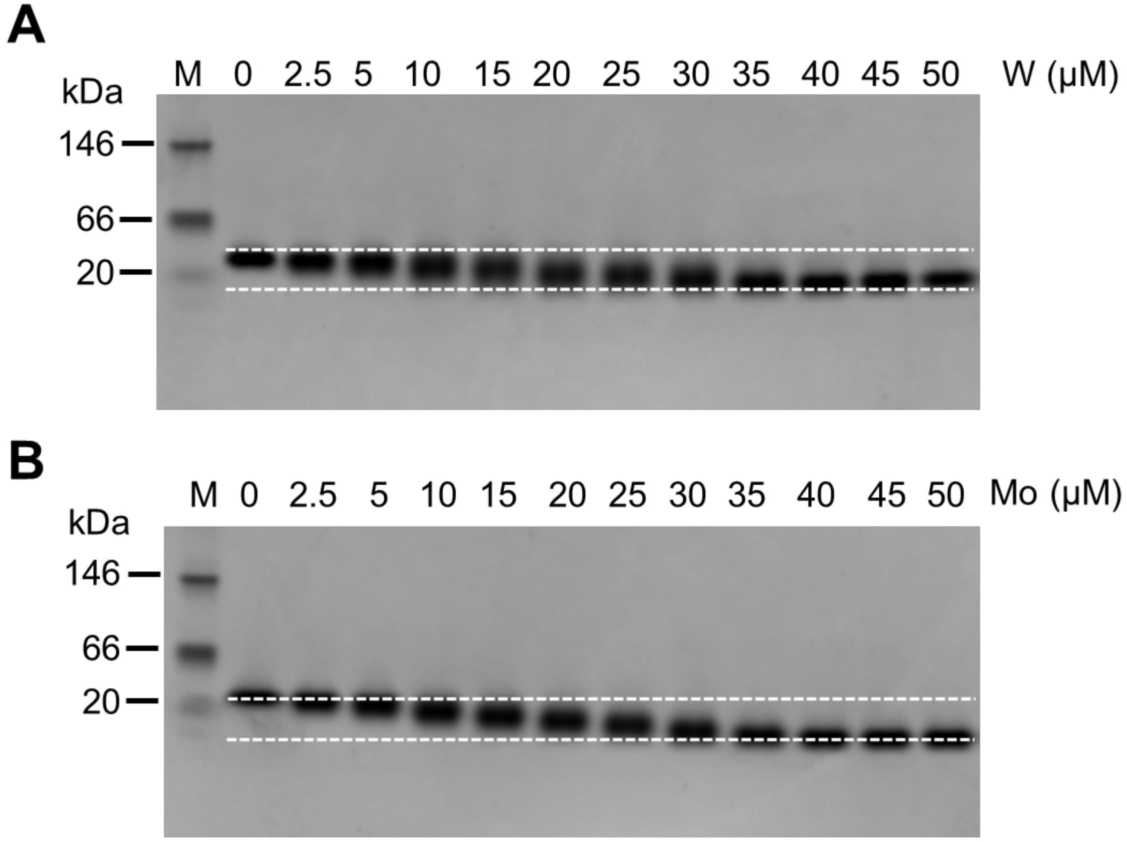


**Fig. S3. Native gel-shift assay with 5 μM hexameric Tub with different W (A) and Mo (B).** Numbers on the wells indicate the W or Mo concentrations incubated with 5 μM hexameric Tub. Purified Tub aliquots were incubated on ice for 1 h with each indicated concentration of anions. The incubated samples were mixed with the native loading buffer (Bio-Rad) and loaded onto a 7.5% native polyacrylamide gel (Bio-Rad). The gel was run in a buffer containing 25 mM Tris and 192 mM glycine at 4 °C for 75 minutes at 100 V, and then stained with AcquaStain (Bulldog Bio).

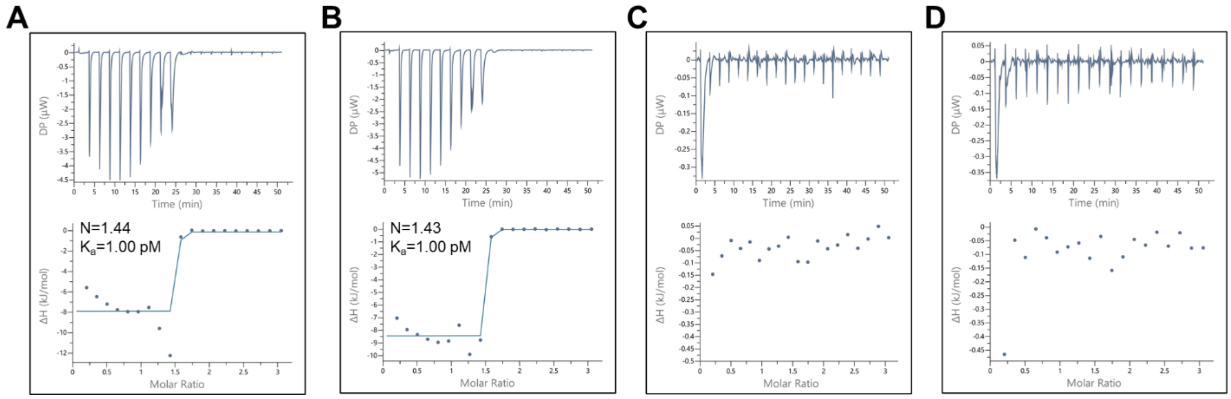


**Fig S4. Isothermal titration calorimetry (ITC) profiles for the binding of tungstate or molybdate by Tub.** ITC involved titrating 50 µM hexameric Tub with 6 mM tungstate (A) or molybdate (B) and subsequently titrating 6 mM tungstate (C) or molybdate (D) into 300 µM molybdate or tungstate saturated Tub. Calorimetric measurements were performed at 25 °C using a MicroCal PEAQ-ITC (Malvern Panalytical, Malvern, United Kingdom). Tub (50 µM hexamer) in 25 mM Tris, pH 7.6, 100 mM NaCl (ITC buffer in the sample chamber (300 µL) was titrated with 6 mM $Na_2WO_4$ or 6 mM $Na_2MoO_4$ dissolved in the same ITC buffer to achieve a final molar ratio of oxyanion to monomeric Tub of 3.5:1. Data were analyzed using Malvern MicroCal PEAT-ITC analysis software (Malvern Panalytical, Malvern, United Kingdom) (2).

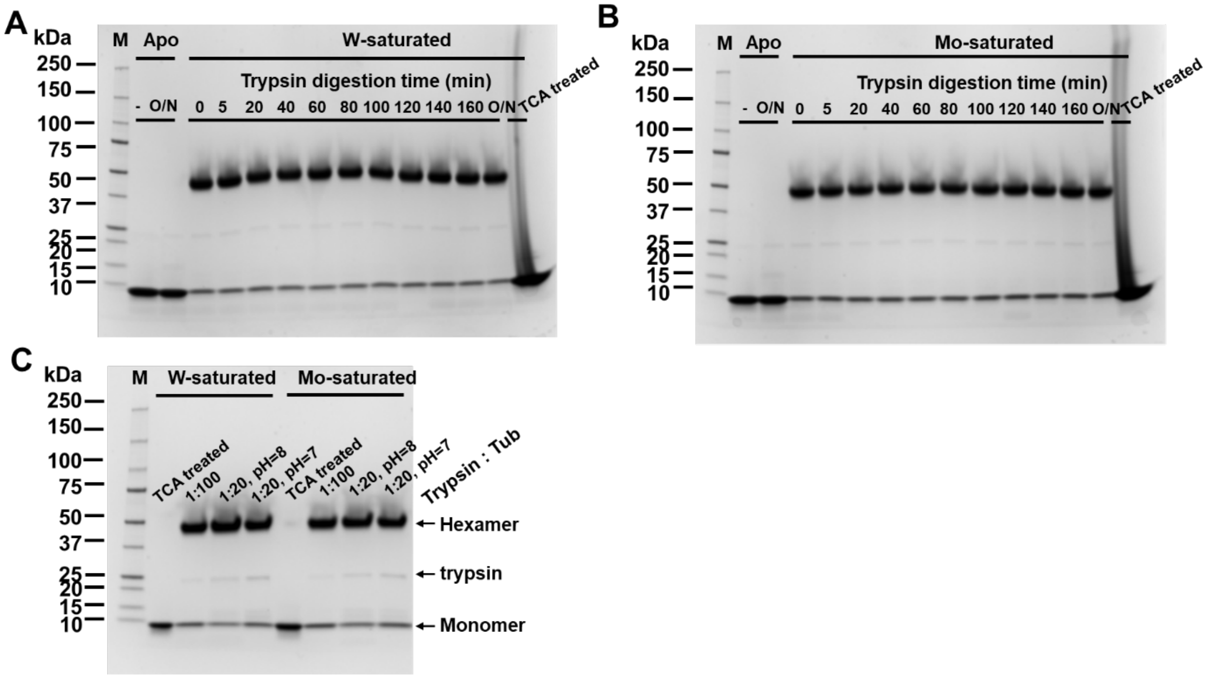


**Fig. S5. Tub resists trypsin digestion but undergoes denaturation upon TCA precipitation.** Purified rTub was diluted to 0.5 μg/μL in 20 μL of 25 mM Tris, pH 7.6, 100 mM NaCl. Trypsin was added at a ratio of 1:20 or 1:100 (trypsin-to-protein) and the digestion proceeded for periods of 5 to 160 min at 37 °C, or overnight. Each resulting mixture was denatured by heating at 98 °C for 10 minutes to inactivate the trypsin, then mixed with loading buffer, and loaded onto an SDS-PAGE gel. SDS-PAGE analysis of Tub with full W saturation (*A*) or Mo saturation (*B*) after trypsin treatment. Different trypsin digestion conditions, including at pH 7.0 and pH 8.0, and trypsin-to-Tub ratios of 1:100 and 1:20, were also evaluated (*C*). Tub samples were treated with trypsin at a ratio of 1:100 (trypsin/Tub, w/w) at 37 °C, pH 8, for the time indicated above each lane, including overnight (O/N). TCA treated indicates Tub treated with trichloroacetic acid and acetone, loaded without trypsin digestion. Molecular weights based on standards are labeled on the left.

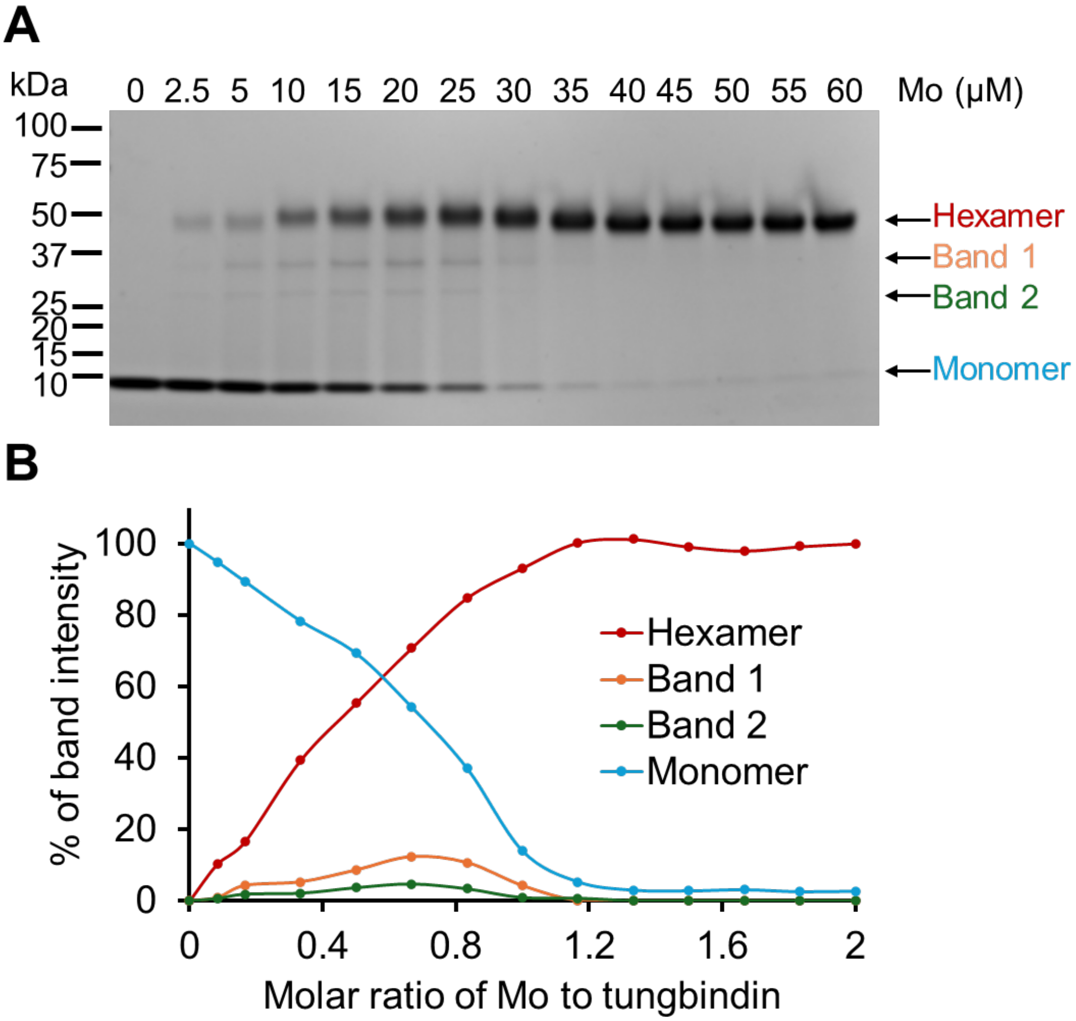


**Fig. S6. The hexameric form of Tub exhibits high thermal stability with Mo bound.** The purified recombinant Tub was diluted to 5 μM in hexamer within a buffer containing 25 mM Tris, pH 7.6, 100 mM NaCl. The 0-60 μM molybdate ($MoO_4^{2-}$) was added to Tub aliquots and incubated on ice for 1 h with each indicated concentrations of anions. The samples were mixed with loading buffer, denatured by heating at 98 °C for 10 minutes and loaded onto precast 4-15% SDS-PAGE gels (Bio-Rad). The gel was run in a buffer containing 25 mM Tris, 192 mM glycine, 0.1% SDS at room temperature for 20 minutes at 200 V, and then stained with AcquaStain (Bulldog Bio). (A) SDS-PAGE analysis showed the hexametric form of Tub remains intact upon heating after binding Mo. Molecular weights based on standards are labeled on the left. (B) The relative intensity of hexamer (red), intermediate band 1 (orange), intermediate band 2 (green), and monomer (blue) in each well estimated using ImageJ (https://imagej.nih.gov/ij/).

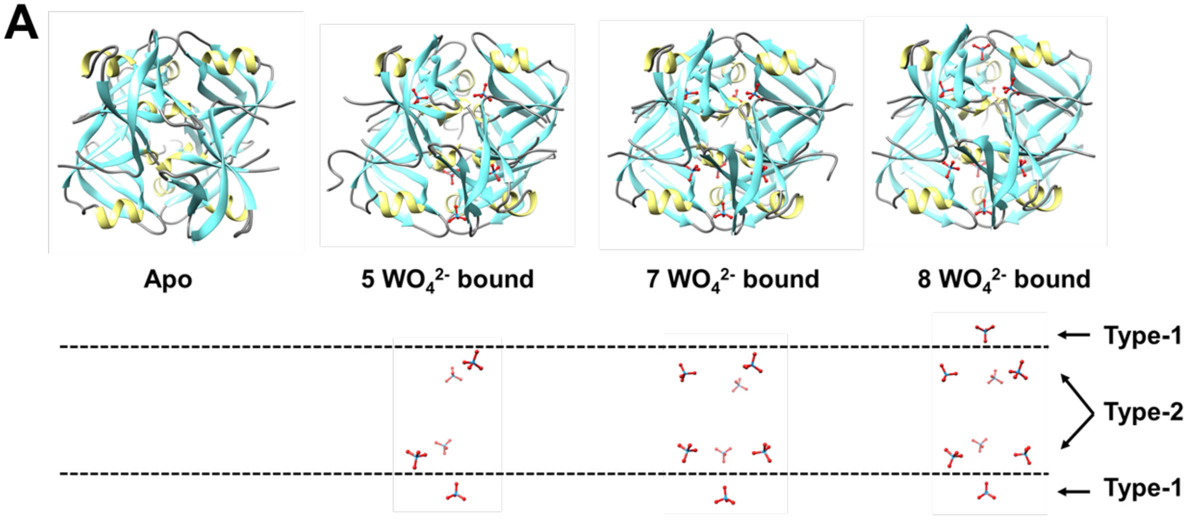


**B**

| | Sites | 5 $WO_4^{2-}$ bound | | 7 $WO_4^{2-}$ bound | | 8 $WO_4^{2-}$ bound | |
|---|---|---|---|---|---|---|---|
| | | **Occupancy** | **B-factor** | **Occupancy** | **B-factor** | **Occupancy** | **B-factor** |
| Type-1 sites (1-2) | $WO_4^{2-}$-1 | 0.71 | 67.01 | 0.60 | 41.34 | 1.0 | 20.12 |
| | $WO_4^{2-}$-2 | | | | | 1.0 | 20.60 |
| Type-2 sites (3-8) | $WO_4^{2-}$-3 | 0.72 | 42.37 | 0.80 | 33.18 | 1.0 | 19.73 |
| | $WO_4^{2-}$-4 | 0.67 | 45.86 | 0.78 | 34.89 | 1.0 | 19.56 |
| | $WO_4^{2-}$-5 | | | 0.68 | 30.56 | 1.0 | 18.72 |
| | $WO_4^{2-}$-6 | 0.66 | 76.81 | 0.75 | 33.36 | 1.0 | 18.36 |
| | $WO_4^{2-}$-7 | 0.63 | 61.89 | 0.71 | 33.25 | 1.0 | 19.12 |
| | $WO_4^{2-}$-8 | | | 0.73 | 36.47 | 1.0 | 20.20 |

**Fig. S7. Ligand binding states of Tub.** (*A*) Crystal structures of Tub with 0, 5, 7, and 8 $WO_4^{2-}$ bound, along with their respective occupied binding sites shown below the structures. (*B*) Table summarizing the occupancy and B-factor ($Å^2$) statistics for $WO_4^{2-}$ in binding sites after structure solution and structure refinement.

A

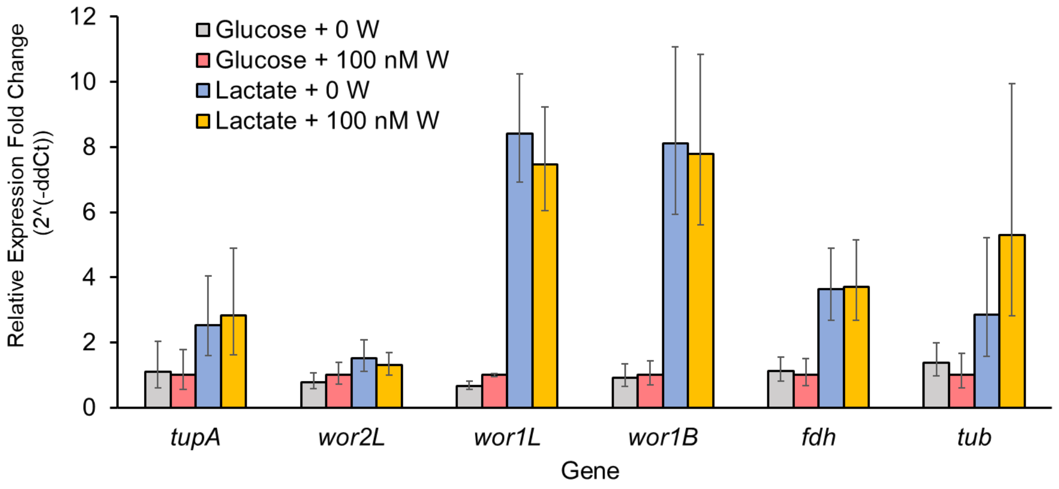


B

| Growth condition comparison | *tupA* | *wor2L* | *wor1L* | *wor1B* | *fdh* | *tub* |
|---|---|---|---|---|---|---|
| Glucose + 0 W vs Glucose + 100 nM W | 0.776 | 0.233 | 0.023* | 0.742 | 0.532 | 0.185 |
| Glucose + 0 W vs Lactate + 0 W | 0.044* | 0.016* | < 0.0001**** | 0.0003*** | 0.0003*** | 0.061 |
| Glucose + 100 nM W vs Lactate + 100 nM W | 0.031* | 0.150 | < 0.0001**** | 0.0004*** | 0.002** | 0.848 |
| Lactate + 0 W vs Lactate + 100 nM W | 0.684 | 0.345 | 0.505 | 0.813 | 0.839 | 0.268 |

**Fig. S8. Quantitative real-time PCR analysis of relative gene expression changes in *E. limosum*.** (A) *E. limosum* cultures (50 mL) were grown in 160-mL serum bottles on glucose (gray/red) and lactate (blue/yellow), with and without added 100 nM tungstate, respectively, until they reached early log phase ($OD_{600}$~0.3). Cultures were chilled on ice for 10 min and cells were then harvested by centrifugation at 10,000 × g at 4 °C for 20 min. The RNA extraction and qRT-PCR were performed as described (3). cDNA synthesis was performed using 0.1 μg purified RNA with the Affinity Script QPCR cDNA synthesis kit (Agilent). Quantitative RT-PCR was conducted using Brilliant II SYBR Green QPCR Master Mix (Agilent). Primers were designed to amplify ~ 150 bp product within the target gene. The locus tags for genes *tupA*, *wor2L*, *wor1L*, *wor1B*, *fdh*, and *tub* are B2M23_RS13650, B2M23_RS04635, B2M23_RS16040, B2M23_RS16055, B2M23_RS12615, B2M23_RS18545, respectively. The gene encoding DNA topoisomerase (ATP-hydrolyzing) subunit B (gyrB, B2M23_RS03195) was used as the reference gene. Error bars represent standard deviations. (B) Table of *p*-values for comparisons of each growth condition. **p* value ⩽ 0.05, ***p* value ⩽ 0.01, ****p* value ⩽ 0.001, *****p* value ⩽ 0.0001.

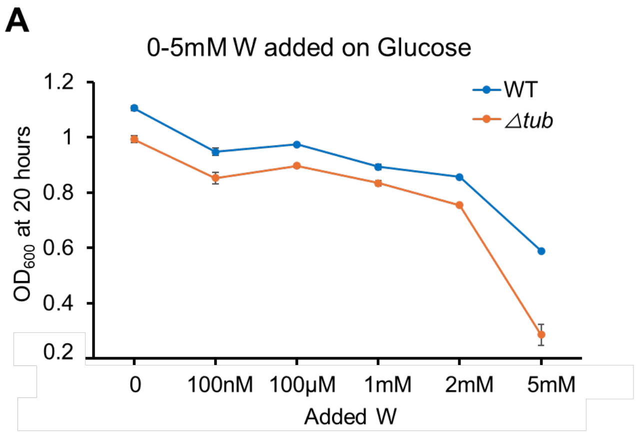


**Fig. S9. Comparison of the cell density ($OD_{600}$) between *E. limosum* wild type and Δ*tub* mutant cultured for 20 hours on glucose (A) or for 60 hours on lactate (B).** $OD_{600}$ values were recorded with the indicated tungstate concentration added to the growth media. Error bars represent standard deviations. When no W is added, the medium contains approximately 5 nM W as a contaminant from other chemicals that are present (4).

**Table. S1. Proteins identified by LC-MS/MS in the ~7kDa SDS gel band of fraction 16 from SEC column.** This is supplied as an Excel file.

**Table. S2. Proteins identified by LC-MS/MS in fraction 16 from SEC column.** This is supplied as an Excel file.

**Table S3. Crystallization conditions for Tub.**

| Protein name | Crystallization condition | Cryo-protectant |
|---|---|---|
| Tub (8 $WO_4^{2-}$ bound) | 0.1 M Magnesium acetate, 0.1 M Sodium cacodylate pH 6.5, 15%(w/v) PEG 6000 | 15% glycerol |
| Tub (7 $WO_4^{2-}$ bound) | 0.2 M Ammonium sulfate, 0.1 M MES pH 6.5, 20%(w/v) PEG 8000 | N/A |
| Tub (5 $WO_4^{2-}$ bound) | 0.2 M Ammonium citrate tribasic pH 7.0, 20% w/v Polyethylene glycol 3,350 | N/A |
| Apo-Tub | 1.1 M Sodium malonate pH 7.0, 0.1 M HEPES pH 7.0, 0.5% v/v Jeffamine ® ED-2001 pH 7.0 | 10% ethyl glycol |

**Table S4. Data collection and refinement statistics. All data sets were collected using CuKα radiation (λ=1.5418 Å).**

| | Tub (8 $WO_4^{2-}$ bound) | Tub (7 $WO_4^{2-}$ bound) | Tub (5 $WO_4^{2-}$ bound) | Apo-Tub |
|---|---|---|---|---|
| **Data collection** | | | | |
| Space group | P 43 21 2 | P 43 21 2 | P 43 21 2 | P 43 21 2 |
| Cell dimensions | | | | |
| *a*, *b*, *c* (Å) | 74.81 74.81 156.31 | 74.75 74.75 156.58 | 74.35 74.35 148.83 | 75.44 75.44 248.69 |
| α, β, γ (°) | 90 90 90 | 90 90 90 | 90 90 90 | 90 90 90 |
| Resolution (Å) [a] | 39.08 - 1.85 (1.87 -1.85) | 43.81 - 2.26 (2.29 - 2.26) | 42.88 - 2.68 (2.74 - 2.68) | 47.98 - 2.89 (2.94 - 2.89) |
| $R_{merge}$ | 0.098 (0.530) | 0.122 (0.577) | 0.173 (1.018) | 0.177(0.788) |
| $I$ / σ | 36.26(3.20) | 31.30 (2.94) | 31.75 (3.75) | 9.80 (1.97) |
| Completeness (%) | 97.2 (69.2) | 95.3 (68.2) | 99.9 (99.0) | 99.1 (97.8) |
| Redundancy | 34.1 (2.9) | 16.3 (4.3) | 22.9 (17.0) | 7.7 (6.3) |
| **Refinement** | | | | |
| Resolution (Å) | 1.85 | 2.26 | 2.68 | 2.89 |
| No. reflections | 36536 (888) | 20521 (482) | 12180 (724) | 16623 (713) |
| $R_{work}$ / $R_{free}$ | 0.1687/0.1888 | 0.1782/0.2293 | 0.2013/0.2579 | 0.2839/0.3226 |
| No. atoms | 3413 | 3419 | 3187 | 3016 |
| Protein | 2984 | 3004 | 3006 | 2948 |
| Ligand/ion | 40 | 40 | 45 | 40 |
| Water | 389 | 375 | 136 | 28 |
| *B*-factors | 22.50 | 27.95 | 42.90 | 39.66 |
| R.m.s. deviations | | | | |
| Bond lengths (Å) | 0.009 | 0.010 | 0.010 | 0.010 |
| Bond angles (°) | 0.98 | 1.22 | 1.25 | 1.37 |
| Ramachandran | | | | |
| favored | 99.26 | 99.01 | 98.03 | 99.00 |
| allowed | 0.74 | 0.99 | 1.23 | 1.00 |
| outliers | 0.00 | 0.00 | 0.74 | 0.00 |
| PDB accession code | 9BEB | 9BEM | 9BEL | 9BJF |

[a] Highest resolution shell is shown in parentheses.

**Table S5. The number of proteins, genomes and species representatives by TOBE domain architecture in the human gut microbiome.** All architectures detected in the UHGP-90 protein clustered are reported. Here we have attempted to separate the canonical Mop family proteins, proteins with exactly one TOBE-domain that have sequence length <= 75 amino acids, and those that contain exactly one TOBE-domain but appear to be significantly longer. The 510 clustered protein representatives were mapped back to the originating proteins and their genomes and then to their subsequent species representatives. The differences in totals at the bottom are reflection grouping genome and species representatives by architectures, as opposed to just counting any TOBE-containing protein. In short, this difference means many genomes, and by association some species representatives, have multiple TOBE-domain containing proteins.

| Functional Group | Family Name (*Proposed Name) | TOBE (IPR005116) Domain Architecture | Number of Proteins | Percentage of All TOBE-Containing Proteins | Number of Genomes | Number of Species Rep. | Percentage of Species Rep. |
|---|---|---|---|---|---|---|---|
| Storage | Tub/Mop | **IPR005116** (<=75 aa) | 5,660 | 12% | 5,527 | 216 | 23% |
| | *Long-Tub/Mop | **IPR005116** (>75 aa) | 2,449 | 5% | 2,404 | 119 | 13% |
| | TudG/ModG | **IPR005116**,**IPR005116** | 5,379 | 11% | 5,348 | 148 | 16% |
| Transport | TupC/ModC | IPR003439,**IPR005116** | 13,330 | 28% | 13,294 | 197 | 21% |
| Regulation | *TudE/ModE-like | IPR000847,**IPR005116** | 5,519 | 12% | 5,461 | 100 | 11% |
| | TudE/ModE | IPR000847,**IPR005116**,**IPR005116** | 8,642 | 18% | 8,637 | 87 | 9% |
| | *TaoR-like | **IPR005116**,IPR041657,IPR024370 | 3,818 | 8% | 1,975 | 32 | 3% |
| Uncommon | *ATP2-Tub/Mop (possible TupB/ModB) | IPR000515,IPR003439,**IPR005116** | 26 | < 1% | 26 | 9 | 1% |
| | *ATP3-Tub/Mop | IPR003439,**IPR005116**,IPR006132,IPR006131 | 1,729 | 4% | 921 | 7 | 1% |
| | *MerR-Tub/Mop | IPR041657,**IPR005116** | 5 | < 1% | 5 | 3 | < 1% |
| | *TXN-Tub/Mop | IPR012336,**IPR005116** | 379 | 1% | 216 | 3 | < 1% |
| | *AaeB-Tub/Mop | IPR006726,**IPR005116** | 26 | < 1% | 26 | 2 | < 1% |
| | *ATP4-Tub/Mop (possible TupC/ModC) | IPR003439,IPR040582,**IPR005116** | 2 | < 1% | 2 | 2 | < 1% |
| | *TudG/ModG-like | **IPR005116**,**IPR005116**,**IPR005116** | 16 | < 1% | 16 | 1 | < 1% |
| | *Lipid-Tub/Mop | IPR010511,IPR008258,**IPR005116** | 5 | < 1% | 5 | 1 | < 1% |
| | *DUF-Tub/Mop | IPR032491,**IPR005116** | 1 | < 1% | 1 | 1 | < 1% |
| | | **Total as Classified by TOBE (IPR005116) Domain Architecture** | **46,986** | **100%** | **43,864** | **928** | **100%** |
| | | **Total of All TOBE (IPR005116) Containing** | **46,986** | **100%** | **25,608** | **559** | **100%** |

**Table S6. TOBE domain architectures in all protein sequence space**. All architectures with an abundance of at least 1% based on InterPro website as of May 2024. The representative length is calculated by InterPro.

| Functional Group | Proposed Family Name | TOBE (IPR005116) Domain Architecture | Rep. AA Length | Number of Proteins | Percentage of All TOBE Containing Proteins |
|---|---|---|---|---|---|
| Storage | Tub/Mop | **IPR005116** (<= 75 aa + >75 aa) | 68 | 7,862 | 19% |
| | TudG/ModG | **IPR005116**,**IPR005116** | 142 | 3,409 | 8% |
| Transport | TupC/ModC | IPR003439,**IPR005116** | 344 | 14,972 | 35% |
| Regulation | TudE/ModE-like | IPR000847,**IPR005116** | 255 | 1,067 | 3% |
| | TudE/ModE | IPR000847,**IPR005116**,**IPR005116** | 270 | 2,861 | 7% |
| Other | ATP4-Tub/Mop (possible TupC/ModC) | IPR003439,IPR040582,**IPR005116** | 377 | 6,641 | 16% |
| | MerR-Tub/Mop | IPR041657,**IPR005116** | 130 | 2,928 | 7% |
| | MerR2- Tub/Mop | IPR000551,**IPR005116** | 131 | 1,519 | 4% |
| | ATP2-Tub/Mop (possible TupB/ModB) | IPR000515,IPR003439,**IPR005116** | 621 | 603 | 1% |

**Table S7. The number of TOBE-domain containing proteins in same genomes and species representatives in the human gut microbiome.** All the proteins reported in Supp Table 5 were grouped by frequency within a genome. These data include genomes that have two or more of the exact same domain architecture in the same genome. For example, *E. limosum* has only one TOBE-containing protein, Tub, and would be represented by row #1. *For species representative the maximum number of proteins for each genome was counted for the grouping. For example, of the 290 species representative with one protein, not one genome contained more than one protein.

| Number TOBE Proteins in a Genome | Number of Proteins | Number of Genomes | Percent of Genomes | Number of Species Rep.* | Percent of Species Rep.* |
|---|---|---|---|---|---|
| 1 | 8831 | 8831 | 34% | 290 | 52% |
| 2 | 24722 | 12361 | 48% | 165 | 30% |
| 3 | 12768 | 4256 | 17% | 86 | 15% |
| 4 | 552 | 138 | 1% | 12 | 2% |
| 5 | 95 | 19 | < 1% | 4 | 1% |
| 6 | 18 | 3 | < 1% | 2 | < 1% |
| **Total** | **46986** | **25608** | **100%** | **559** | **100%** |

**Table S8. The co-occurrence of tungsten- and molybdenum-related proteins in species representatives in the human gut microbiome.** Data is represented as a table with percentage and a Venn diagram for clarity of co-occurrence. Some species representatives are counted more than once per table because some genome members are classified into different groups. For example, there 403 species representatives with at least one WOR containing member (data not shown) but there are 490 WOR species representatives listed below. Likewise, there are 821 ModA species representatives and 282 TupA species representatives with at least one ModA or TupA containing member (data not shown).

| Protein Groups | Number of Species Representatives | Percentage of Species Representatives |
|---|---|---|
| ModA | 551 | 54% |
| WOR | 242 | 24% |
| TupA | 107 | 11% |
| TupA, WOR | 96 | 9% |
| ModA, WOR | 89 | 9% |
| TupA, ModA | 63 | 6% |
| TupA, ModA, WOR | 63 | 6% |
| **Total** | **1015** | **100%** |

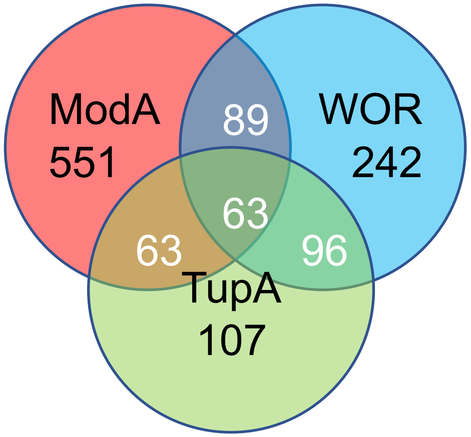

**Table S9. TOBE domain architectures identified in species representatives classified their predicted primary physiological metal.** Combining the data in Table S5 and Table S8, we used the presence or absence of W- and/or Mo-related genes to predict the physiological metal (W or Mo) that various TOBE proteins bind in a given genome then ultimately a species representative (Fig. 6). The four primary physiological metal categories are: likely to bind W (W), could bind either W or Mo (Either), likely to bind Mo (Mo), or are unknown to bind anything (Unknown). A W-binding TOBE is predicted when TupA is present in a member genome of a species representative, a Mo-binding TOBE is predicted when only ModA is present, and a W- or Mo-binding is predicted when TupA is absent but WOR is present. Unknowns are species representatives where no member genome is predicted to contain TupA or ModA. ♦Since any one species representatives in the table has a one-to-many (e.g., 1:1000) relationship with the member genomes (Fig. 5); and each member genome can have a different (and multiple) TOBE domain architectures to W- and/or Mo-related gene combinations, thus some species representatives can be counted multiple times in a row, column, or table total. ♦♦In an effort estimate the overcount of species representatives per row, column and table (and preserve to the member genome information), a distinct (or single occurrence) species representatives count per row, column and table is provided. The difference between these totals provides some insight into the member genome variation present in species representatives.

| Functional Group | Family Name (*Proposed Name) | TOBE (IPR005116) Domain Architecture | W: TupA, WOR | W: TupA, ModA, WOR | W: TupA Only | W: TupA, ModA | Either: WOR | Either: ModA, WOR | Mo: ModA | Unknown: None | ♦Sum of Species Reps. (multiple- occurrence ) | ♦♦Distinct Sum of Species Reps. (single-occurrence) |
|---|---|---|---|---|---|---|---|---|---|---|---|---|
| | | | Predicted Primary Physiological Metal | | | | | | | | | |
| | Tub/Mop | **IPR005116** (<=75 aa) | 6 | 9 | 4 | 9 | 20 | 17 | 71 | 116 | | |
| Storage | *Long-Tub/Mop | **IPR005116** (>75 aa) | 8 | 5 | 5 | 16 | 22 | 17 | 26 | 59 | 613 | 483 |
| | TudG/ModG | **IPR005116**,**IPR005116** | 14 | 17 | 7 | 19 | 21 | 18 | 46 | 61 | | |
| Transport | TupC/ModC | IPR003439,**IPR005116** | 1 | | 2 | 7 | 29 | 15 | 85 | 95 | 234 | 197 |
| | *TudE/ModE-like | IPR000847,**IPR005116** | 1 | | | 4 | 16 | 4 | 32 | 57 | | |
| Regulation | TudE/ModE | IPR000847,**IPR005116**,**IPR005116** | | | 1 | 3 | 14 | 5 | 40 | 38 | 252 | 219 |
| | *TaoR-like (Tub/Mop-MerR-TupA) | **IPR005116**,IPR041657,IPR024370 | 3 | 1 | 1 | | 2 | | 4 | 26 | | |
| | *ATP2-Tub/Mop (possible TupB/ModB) | IPR000515,IPR003439,**IPR005116** | | | | | | | 7 | 4 | | |
| | *ATP3-Tub/Mop (Asp/Orn binding) | IPR003439,**IPR005116**,IPR006132,IPR006131 | | | | | 3 | 3 | 2 | 5 | | |
| | *MerR-Tub/Mop | IPR041657,**IPR005116** | | | | | | 2 | 2 | | | |
| | *TXN-Tub/Mop | IPR012336,**IPR005116** | | | | | 1 | | | 3 | | |
| Uncommon | *AaeB-Tub/Mop | IPR006726,**IPR005116** | | | | | | | | 2 | 39 | 29 |
| | *ATP4-Tub/Mop (possible TupC/ModC) | IPR003439,IPR040582,**IPR005116** | | | | | | | 1 | 1 | | |
| | *TudG/ModG-like | **IPR005116**,**IPR005116**,**IPR005116** | | | | | | | | 1 | | |
| | *Lipid-Tub/Mop | IPR010511,IPR008258,**IPR005116** | | | | | | | | 1 | | |
| | *DUF-Tub/Mop | IPR032491,**IPR005116** | | | | | 1 | | | | | |
| | | ♦Sum of Species Reps. (multiple- occurrence ) | 33 | 32 | 20 | 58 | 129 | 81 | 316 | 469 | 1138 | 928 |
| | | ♦♦Distinct Sum of Species Reps. (single-occurrence) | 23 | 28 | 17 | 37 | 66 | 51 | 195 | 283 | 700 | 559 |

**Table S10. A complete list of UHGG genes, genomes and species representative that encode a TOBE domain protein, and their predicted W- and Mo-related proteins based on InterPro and EggNOG.**

This is supplied as an Excel file.

**Table S11. Primers used in this study.**

| Primers | Sequence (5'-3') |
|---|---|
| Tub-F | GAGATATACATATGAAATTAAGTGCTCGC |
| Tub-R | CCGGATCTCACTATTTTTCGAACTGCGGGTGGCTCCAGTCATCAATACCGATC |
| pET24-F | TCGAAAAATAGTGAGATCCGGCTGCTAAC |
| pET24-R | TTAATTTCATATGTATATCTCCTTCTTAAAGTTAAACAAAATTATTTC |
| Up Tub-F | CTACGCATTCAGTAGAAATCAAAAAAATGGAG |
| Up Tub-R | AAACCACAAAAAGAAATGCCTTCTTTCTATG |
| ermB-F | GGCATTTCTTTTTGTGGTTTATTTACAAATTCGG |
| ermB-R | TTTTTTAATATGTTTGCAAGCAGCAGATTAC |
| Dn Tub-F | CTTGCAAACATATTAAAAAAACCAGGACGCTTTTAAC |
| Dn Tub-R | TTCGCTCCGGACAATTGC |

## References


1. Rech S, Wolin C, Gunsalus RP. 1996. Properties of the periplasmic ModA molybdate-binding protein of *Escherichia coli*. J Biol Chem 271:2557-62.
2. Ge X, Thorgersen MP, Poole FL, 2nd, Deutschbauer AM, Chandonia JM, Novichkov PS, Gushgari-Doyle S, Lui LM, Nielsen T, Chakraborty R, Adams PD, Arkin AP, Hazen TC, Adams MWW. 2020. Characterization of a metal-resistant *Bacillus* strain with a high molybdate affinity ModA from contaminated sediments at the Oak Ridge Reservation. Front Microbiol 11:587127.
3. Thorgersen MP, Schut GJ, Poole FL, 2nd, Haja DK, Putumbaka S, Mycroft HI, de Vries WJ, Adams MWW. 2022. Obligately aerobic human gut microbe expresses an oxygen resistant tungsten-containing oxidoreductase for detoxifying gut aldehydes. Front Microbiol 13:965625.
4. Schut GJ, Thorgersen MP, Poole FL, 2nd, Haja DK, Putumbaka S, Adams MWW. 2021. Tungsten enzymes play a role in detoxifying food and antimicrobial aldehydes in the human gut microbiome. Proc Natl Acad Sci U S A 118 (43):e2109008118.
